# 2021 World of Shipping Portugal: An International Research Conference on Maritime Affairs Editorial

**DOI:** 10.1186/s41072-021-00099-x

**Published:** 2021-10-28

**Authors:** Ana Cristina Paixão Casaca, Maria Amélia Ramos Loja

**Affiliations:** 1 World of Shipping Portugal, Parede, Portugal; 2 CIMOSM, ISEL - Centro de Investigação Em Modelação E Optimização de Sistemas Multifuncionais (in English: Multifunctional Systems Modelling and Optimization Research Centre), Instituto Politécnico de Lisboa, 1959-007 Lisbon, Portugal; 3 Universidade de Évora, 7000-671 Évora, Portugal; 4 NOVA UNIDEMI, Universidade NOVA de Lisboa, Campus de Caparica, 2829-516 Caparica, Portugal

**Keywords:** Maritime transport, Ports, Innovation management, Evolutionary path, Future of the industry

## Abstract

Currently, the shipping industry is at a crossroads. Although it has overcome numerous technological barriers and finance and economic crises over the years, the industry is facing its most prominent challenge, which rests on finding the most feasible solutions to deal with industry decarbonisation until 2050. Within this scope, the current Editorial addresses the issue of sulphur emissions that the industry faced with the entrance of the sulphur regulation on 1 January 2020 and draws attention to the road that the industry players need to cover to decarbonise the shipping industry. Innovative measures like the Poseidon Principles or the Sea Cargo Charter are in place, and industry players are coming together to find feasible solutions. Within this regulatory environment, the shipping industry also had to deal with the COVID-19 Pandemic. However, some market segments, such as the container and dry bulk ones, have managed to survive, which is not the case with the tanker market. Altogether, these events draw the industry to deal with the market, technology, and regulatory challenges and risks whose outcome is yet to be seen. The Editorial concludes by presenting briefly the papers published in this Special Issue, which were selected among the ones presented at the 2021 World of Shipping Portugal, an international research conference on maritime affairs, 28–29 January 2021, that took place online from Portugal to the World due to the Covid-19 pandemic.

The historical and the current environment in which shipping operates has been a complex and volatile one that needs to adjust to the many boom and bust cycles of the global economy. Adding to this complexity, the International Maritime Organisation (IMO) has been issuing a range of international legislative measures related to maritime safety, marine oil pollution, seafarers’ training, among others, to regulate the industry’s operating environment. However, while they improved the industry’s overall performance, they challenged several industry players to adopt strategies, policies and measures to implement them, usually at a high cost.

As a response to existing environmental concerns, other than oil pollution, such as noxious liquid substances, sewage, garbage, and invasive species from ballast water, the IMO has been releasing various legislative measures to mitigate the impact shipping operations have on the environment. However, the world focus on climate change has also drawn the attention of the IMO. Therefore, in response to existing environmental concerns related to the emission of harmful emissions from ships and improving the global air quality, the IMO issued a regulation to impose new emissions standards designed to curb the global fleet’s pollution significantly. As of 1 January 2020, the IMO banned shipping vessels from using fuel with a higher than 0.5% sulphur content, thus lowering the previous 3.5% limit. Such a high sulphur content harms human health and is a component of acid rain, harming vegetation and aquatic species. Moreover, the IMO extended the existing 0.1% sulphur cap in designated emission control areas worldwide with this regulation.

While restrictions on sulphur emissions in shipping were not entirely new because emissions control areas did exist in some areas of the World, its transition was a daunting challenge to a certain extent. The maritime industry had to stop burning high-sulphur fuels, as required by the IMO and stood in a position where it had to choose among (1) the installation of scrubbers, whose cost ranges from $2 and $10 million for each ship, to go on burning high-sulphur fuel oil, (2) the payment for higher low-sulphur fuels price or (3) the use of alternative ‘cleaner’ fuel-powered vessels, in particular LNG and methanol. Subject to the existing alternatives, it was not surprising that, as of the beginning of July 2019, only 4% of the world fleet was scrubber-fitted and ready to be in operation. The forecasts were not very positive, although this figure was expected to rise to 11% and 15% of the world fleet by the end of 2019 and 2020, respectively.

Moreover, the shipping industry faced a range of uncertainties relating to the actual impact of the regulation. For example, uncertainties related to (1) the reliability of the scrubber technology; (2) the capacity of refineries in being able to supply the worldwide shipping market, as vessels switch from high- to low/very low-sulphur fuel oil; (3) the worldwide distribution of low-sulphur fuels which raised concerns about their availability in small or distant ports; (4) the price of low/very low-sulphur fuel oil and of high-sulphur fuel oil after 1 January 2020; (5) the contribution that the new regulation brought to the environment; (6) the behaviour of shipping companies in what concerned the definition of their operational and bunkering strategies; and (7) the level of impacts that such a fuel switch had on the shipping industry’s operating costs and global freight rates. The 2020 sulphur regulation was expected to cost the industry about US$50 billion in 2020, and according to a Reuters report, analysts estimated that the container industry alone was expected to bear additional costs of approximately US$10 billion.

However, the entrance of this regulation was just the tip of the iceberg of what was yet to come. The new fuel standards target sulphur emissions, not greenhouse gas emissions. This means that the industry has yet to control greenhouse gas emissions to limit global warming to well below 2 °C above pre-industrial levels by 2050, aiming to limit the temperature increase to 1.5 °C as requested by the Paris Agreement reached in December 2015. According to a November 2019 United Nations Report, the World needs to reduce greenhouse gas emissions by 7.6% per year until 2030 to meet the Paris Agreement’s goals on climate change.

Put all this into perspective, and given the size of the fleet, the road towards decarbonising the shipping industry is a long one. Nevertheless, the industry is taking its first steps, and innovative measures like the Poseidon Principles or the Sea Cargo Charter are in place. While the former establishes a framework for assessing and disclosing the climate alignment of ship finance portfolios, the latter establishes a framework for aligning chartering activities with responsible environmental behaviour to support the industry decarbonisation. Furthermore, many industry players are coming together to identify the most feasible solutions the industry needs for its short and deep-sea operations. Attention is being drawn to the best alternative fuels that can replace traditional fossil fuels. The possibilities lie in liquefied natural gas as a transitional fuel, ammonia and hydrogen. However, the problem with these alternative fuels is their production and, consequently, distribution. As they need a vast amount of natural resources to be produced, which can only be found in some areas of the planet, they require a well-designed logistics plan for moving them from their production sites to their consumer market.

If this was not enough, the shipping industry had yet to deal with the COVID-19 Pandemic. In January 2020, the COVID-19 Pandemic changed the functioning of the overall economy. As we moved into 2020, the coronavirus outbreak forced governments to adopt numerous unprecedented preventive measures to (1) reduce the number of infected people and (2) avoid that the number of human lives being lost increased. However, the implementation of such measures, namely social distancing, wearing face masks in public, hand washing, to name a few, have resulted in global social and economic disruptions. Most of the worldwide population was forced into confinement periods. Teleworking has become the norm, and with it, a climate of social and even emotional disruption/instability. The loss of face-to-face social contact routines was very damaging and, in some cases, actually detrimental to the quality of the work carried out. Many educational institutions were partially or fully closed. Shopping has been limited to the essentials. In many countries, panic buying exacerbated widespread supply shortages. In others, there has been agricultural disruption and food shortages. Events have been cancelled, postponed or transferred online. The market witnessed the emergence of online platforms, among many other technologies.

In April 2020, the International Monetary Fund forecasted global growth in 2020 to fall to − 3%, while the World Trade Organisation projected a world trade to fall by between 13 and 32%, indicating a world recession that many already classify as the most significant global recession since the Great Depression. Furthermore, the COVID-19 Pandemic stressed how nations are globally interdependent, raising questions about the globalisation process witnessed over the last decades, drawing attention to reshoring and nearshoring rather than outsourcing goods from distant locations. Moreover, it has shown that all economic activities, including transport, were utterly unprepared to deal with such public health crisis. Concerning this unpreparedness, the UNCTAD 2020 report draws attention to the need for investing in risk management and emergency response preparedness in transport and logistics.

The COVID-19 Pandemic also impacted the maritime industry. Maritime trade has slowed due to weakening economic growth and trade tensions, and many of the World’s largest ports have reported reductions in the cargo volumes being handled. In November 2020, the UNCTAD estimated that global maritime trade was expected to decrease by 4.1% in 2020 due to the unprecedented disruptions caused by the COVID-19 Pandemic. Thus, the short-term outlook for maritime trade was severe. The pandemic waves prevented foreseeing when the industry recovered even though UNCTAD expected maritime trade growth of 4.8% in 2021, assuming a world economic recovery. However, the stimulus money authorised by the American Congress into the economy during the pandemic promoted a change in the maritime sector, particularly in the container market segment due to the intensive buying performed by the American households. With more goods to be carried by sea, the container shipping market witnessed a boom due to increased demand for shipping services, resulting in severe operational constraints beyond its control. The US ports’ inability to deal with this increase in demand, to which should be added the lack of hinterland connectivity and the numerous COVID-19 cases that affected port labour in the US and China, have resulted in serious port congestions all over the World and consequently supply chain disruptions. If this were not sufficient, the temporary blockade of the ultra-large container ship “Ever Given” in the Suez Canal, forcing some shipping companies to re-route and reschedule their ships voyages, also contributed to supply chains disruptions and delaying empty containers’ reposition from the consumption to production markets.

Within this context, the following papers have been chosen to be part of this Special Issue of 2021.

*Fulconis and Lissillour* deal with classification societies and maritime safety. According to Fulconis and Lissillour, classification societies play a major role in maritime safety and international shipping market regulation. This paper aims to understand better this specific role and its role in international shipping’s inter-organisational dynamics within this scope. To achieve this, the authors use a conceptual framework provided by the behaviourist approach and applied it to supply chains’ inter-organisational dynamics.

*Dominguez-Péry et al.* carry out a systematic literature review to reduce tanker accidents by tackling human error and identify a future research agenda, even though the number of tanker accidents has fallen over the past 30 years. To conduct this systematic literature review, the authors focus on three main aspects of human error, (1) human resources and management, (2) socio-technical Information Systems and Technologies, and (3) cognition-related errors.

*Karaoulanis and Pelagidis* deal with the Panamax market’s behaviour by focusing on expectations and time lags. The authors claim that expectations play a critical role in the freight market for short-term and long-term decision making. The authors investigate the relationship between time lags and time-charter, trip and spot market rates and the Panamax vessels of various ages average earnings. For this, the authors use an autoregressive model has been constructed to perform the statistical analysis.

*Gascón* investigates bareboat charter agreements insurance-related problems. For this, Gascón takes into account the Gard Marine and Energy vs China National Chartering (“The Ocean Victory”) case and highlights the impact of this court decision leading to the release of the standard bareboat charter agreement, the BARECON 2017 form, by the Baltic and International Maritime Council. The paper analyses the new wording of the insurance clause therein and its incidence on the relationship between the parties of both the charter agreement and the insurance contract, as well as the consequences for possible third parties.

Finally, *Olga et al.* focus on green shipping. Olga et al. explore the Greek stakeholders’ intention in accepting and using liquefied natural gas and electricity as alternative fuels. The study clarifies stakeholders’ possible challenges or barriers to the implementation of technology. This research uses a unified model, namely the expansive technology acceptance model in conjunction with the innovation diffusion theory and external variables affecting liquefied natural gas and electricity.

We thank Journal of Shipping and Trade for accepting this Special Issue and China Merchants Energy Shipping for supporting this Special Issue process charges.

Greetings from Lisbon.

Ana Cristina Paixão Casaca and Maria Amélia Ramos Loja.

## Data Availability

Not applicable.

